# Digital and Remote Interventions for Musculoskeletal Aging: Real-Time Muscle Strain Severity Detection Using Artificial Intelligence

**DOI:** 10.3390/bios16070354

**Published:** 2026-06-25

**Authors:** Zulaikha Fatima, Abdullah, Nida Hafeez, Rolando Quintero Téllez, Miguel Jesús Torres Ruiz, Carlos Guzmán Sánchez Mejorada, Miguel Félix Mata-Rivera, Roberto Zagal-Flores

**Affiliations:** 1 Center for Computing Research (CIC), Instituto Politecnico Nacional (IPN), Mexico City 07320, Mexicomtorres@cic.ipn.mx (M.J.T.R.); mmatar@ipn.mx (M.F.M.-R.); rzagalf@ipn.mx (R.Z.-F.); 2 Faculty of Allied Health Sciences, Superior University, Lahore 54000, Pakistan; 3 Department of Computer Science, Bahria University, Lahore 54000, Pakistan

**Keywords:** IoT, machine learning, posture detection, muscle strain severity detection, EMG, NodeMCU ESP8266, Vision Transformer, XGBoost, hybrid model

## Abstract

As global populations grow and technology advances, daily life is increasingly shaped by digital systems such as computers and smart devices. However, prolonged device use has contributed to increasing physical and mental health concerns, particularly those associated with poor sitting posture. Posture-related strain is frequently overlooked and contributes to musculoskeletal discomfort, including back, neck, shoulder, and wrist pain, and may also be associated with sleep disturbances and elevated stress levels. To the best of our knowledge and based on the existing literature, this is the first study to introduce a machine learning-based framework for advanced muscle strain severity classification using Internet of Things (IoT) devices that integrates posture monitoring and muscle strain detection into a unified low-cost framework ($23 hardware cost). The primary objective of this work is accurate classification of muscle strain severity, while real-time alerts serve as a secondary ergonomic feedback mechanism. Specifically, this study makes four major contributions. First, we created a novel dataset through real-time acquisition of electromyography (EMG) and posture signals from participants in hospital and industrial environments, capturing diverse muscle strain patterns validated against clinical assessment procedures. Second, we designed a two-part hardware architecture consisting of posture detection (PD) and strain detection (SD) modules using a NodeMCU ESP8266, HC-SR04 ultrasonic sensor, EMG sensor, and buzzer for real-time physiological monitoring, incorporating EMG-specific preprocessing including band-pass filtering, rectification, and RMS smoothing. Third, we proposed and evaluated a hybrid machine learning framework integrating Vision Transformer (ViT) and XGBoost to classify strain severity into three study-specific categories: baseline (EMG RMS < 40 µV), compensatory strain (40–59 µV), and overload (≥60 µV). These categories were used as reproducible severity proxies for machine learning annotation and should not be interpreted as universal biomarkers of structural tissue damage. Finally, the proposed framework achieved a classification accuracy of 99.0% (95% CI: 98.5–99.5%) with an inference latency of 15.2 ms.

## 1. Introduction

According to the World Health Organization (WHO), 15% of people around the globe live with some form of disability, which accounts for more than 1.3 billion individuals worldwide [1,2]. Among the many contributors to disability, one that is often underestimated is bad posture, especially among office workers and individuals who use digital devices for extended periods [3]. Many individuals sit for long periods without being aware of their posture, which over time leads to numerous health problems [4]. Posture refers to the way a person holds their body while sitting, standing, walking, or performing daily activities. It plays a crucial role in maintaining overall health and well-being. It is recommended, for instance, that individuals maintain a few centimeters distance between their back and the chair while working, as this is considered good sitting posture [5]. However, in today’s digitally driven lifestyle, many people spend hours working with electronic devices without maintaining a proper posture, increasing their risk for musculoskeletal disorders [6].

Unfortunately, public awareness of proper posture is limited. Studies indicate that 53% of individuals are unaware of proper posture, and only 47% understand its negative health impact [7,8]. This limited awareness contributes to common health problems such as back pain, neck pain, shoulder discomfort, wrist pain, sleep disturbances, muscle strain, and even neural complications. These symptoms, when ignored, may progressively worsen and lead to long-term disabilities [9,10]. The problem is not only physical but also psychological. Prolonged physical discomfort due to bad posture can reduce an individual’s focus, vitality, and engagement in work, ultimately impacting productivity. These concerns are even more prominent among older adults, who are more prone to musculoskeletal strain. In the United States, around 80% of consultations are linked to posture-related back and strain issues, with healthcare costs estimated at $45–50 billion annually. [11].

The effects of bad posture on the human body differ significantly, based on factors such as age and their type of intensity of physical activity. Common symptoms include neck pain, back pain, wrist pain, shoulder pain, headaches, sleep disturbances, muscle swelling, redness, loss of strength, and neural disorders [12]. According to various sources, the prevalence of these issues due to poor posture is as follows: back pain at 33.28% [13], muscle swelling at 24.23% [14], neck pain at 13.09% [15], shoulder pain at 9.53% [16], wrist pain at 7.92% [17]. Muscle strain, defined as prolonged or excessive tension in the muscle tissue, is directly impacted by posture and motion. The muscle’s electrical activity reflects the underlying mechanical function that electromyography (EMG) can measure [18]. These electrical signals vary depending on the intensity and duration of mechanical activities like sitting, standing, lifting heavy objects, sleeping in ergonomically improper positions, or repetitive movements, as detected by various sensors [19,20]. Eventually, bad posture may result in serious musculoskeletal disorders beyond superficial pain. The overall effects of bad posture are multifaceted and systemic [21].

According to clinical standards, muscle strain is typically classified into three categories based on its severity: baseline, compensatory strain, and overload [22]. Baseline strain involves limited muscle fiber damage and does not lead to noticeable loss of strength [23]. In compensatory strain, the affected area shows signs of swelling and partial strength loss due to increased contraction. Overload, the most critical category, results in significant muscle damage, including swelling, loss of power, inability to hold objects, and separation between muscle fibers. Muscle strain severity classification follows clinical EMG amplitude thresholds where RMS values below 40 µV indicate baseline activity, 40–59 µV indicate compensatory strain, and ≥60 µV indicate overload, which correlate with the extent of tissue damage [24].

Muscle fatigue and muscle strain represent related but distinct physiological phenomena. Fatigue is generally characterized by progressive reductions in force-generating capability and often manifests through spectral changes in EMG signals, particularly reductions in median frequency and shifts in signal power distribution [25]. In contrast, muscle strain in static occupational settings is frequently associated with sustained elevations in activation amplitude caused by compensatory recruitment patterns during prolonged postural loading [26]. In the present study, strain severity detection focuses primarily on persistent RMS elevation combined with posture context rather than fatigue progression. However, overlap between fatigue and strain may occur during prolonged activities, which represents an acknowledged limitation and an opportunity for future multimodal research [27]. To the best of our knowledge, this is the first IoT-based framework to classify muscle strain severity using EMG biomarkers with a transformer-based architecture in real-world environments.

Recent comprehensive reviews in applied electromagnetic diagnostics highlight the growing role of AI, machine learning, and deep learning in advancing non-invasive assessment and monitoring systems, reinforcing the importance of intelligent sensing frameworks such as the one presented in this work.

We follow a clinical distinction between muscle fatigue and muscle strain to avoid ambiguity. Muscle fatigue refers to a reversible decline in the ability of a muscle to generate force after prolonged or repeated activity and is typically characterized by shifts in spectral EMG features and temporal decline in amplitude during sustained contraction. By contrast, muscle strain denotes structural damage to muscle fibers that varies in severity and is associated with discrete clinical signs (pain, swelling, strength loss) and, in some cases, sustained EMG amplitude elevations linked to compensatory activation patterns. Because our objective is to detect graded strain severity (baseline, compensatory strain, and overload) that reflects potential tissue damage rather than transient fatigue alone, we focus on EMG amplitude-based markers (RMS) and posture context as proxies for strain severity while distinguishing them from classic fatigue metrics such as median frequency shifts. This terminological clarification frames the proposed framework as a strain severity monitoring system rather than a general fatigue detector.

To address this gap, we designed, evaluated, and proposed a novel hybrid model combining a Vision Transformer (ViT) with XGBoost, a machine learning-based framework for advanced muscle strain severity detection using IoT devices. The framework integrates posture detection (PD) and strain detection (SD) modules to continuously monitor an individual’s physical condition in real time. The system is designed to analyze electromyography (EMG) signals and classify muscle strain into clinically relevant categories: baseline, compensatory strain, and overload. The custom hybrid model learns from collected data to improve detection accuracy over time and can distinguish between normal activity and harmful strain patterns. When poor posture is detected alongside compensatory strain or overload, the system triggers a real-time alert via a buzzer and provides real-time data visualization via dynamic graphs on the user’s device. This combination of machine learning with IoT facilitates intelligent, adaptive, and timely feedback for posture correction, significantly reducing the risk of long-term musculoskeletal damage.

This study makes the following major contributions:

- We created a novel real-time dataset by collecting electromyography (EMG) and posture data from participants in university, bank, and industrial environments. During dataset construction, we included a wide range of diverse postural behaviors and muscle strain patterns that can be a valuable resource for training, testing, and validating machine learning models in a dynamic, real-world setting.
- We designed a two-part hardware framework for real-time monitoring of user posture and muscle strain. This framework integrates PD and SD modules; these components enable real-time and accurate detection, providing immediate feedback to assist in the prevention of musculoskeletal damage.
- We proposed and evaluated a custom hybrid model combining Vision Transformer (ViT) and XGBoost, which classifies muscle strain into three clinically meaningful categories—baseline, compensatory strain, and overload—using EMG signals acquired from real-world environments such as banks, offices, and universities.
- The proposed model achieved a reliable accuracy of significantly outperforming baseline models in multi-class muscle strain detection.

Although the proposed system provides real-time alerts through a feedback mechanism, the primary contribution of this work is not intervention itself but reliable classification of muscle strain severity patterns from multimodal physiological and posture information. The alert mechanism acts as a secondary practical extension intended to support user awareness. This distinction is important because the present work focuses on detection performance and signal interpretation rather than evaluating behavioral intervention outcomes.

The remainder of the paper is organized as follows. Section 2 discusses the related work. Section 3 presents the materials and methods, including data collection, annotation, and preprocessing procedures, as well describes the training and testing phases of the machine learning models. Section 4 presents the results, andoutlines the limitations, and Section 5 concludes the paper.

## 2. Related Work

This section reviews existing approaches in posture monitoring, EMG-based muscle analysis, and IoT-driven health systems, highlighting limitations in unified strain severity detection. This section reviews existing approaches in posture monitoring, electromyography (EMG)-based muscle analysis, and IoT-driven health systems, with a focus on identifying limitations in unified muscle strain severity detection.

Yuan et al. [18] introduced GTA-Net, an IoT-enabled 3D pose estimation framework for adolescent sports posture correction. The model integrates Graph Convolutional Networks (GCN), Temporal Convolutional Networks (TCN), and hierarchical attention mechanisms to effectively handle rapid motion, occlusion, and device-level constraints. Evaluated on benchmark datasets including Human3.6M, HumanEva-I, and MPI-INF-3DHP, GTA-Net achieved competitive MPJPE scores of 32.2 mm, 15.0 mm, and 48.0 mm, respectively, demonstrating high accuracy and real-time feedback capabilities. However, its applicability is primarily limited to sports-oriented posture correction and does not extend to physiological strain assessment.

Laidi et al. [19] developed a real-time posture monitoring system using low-cost EMG sensors and Bluetooth Low Energy (BLE) communication integrated with a mobile alert interface. The study evaluated multiple machine learning models, including SVM, K-NN, Decision Tree, Random Forest, and MLP, achieving a maximum classification accuracy of 91% using K-NN. Despite its practical implementation, the study is constrained by a relatively small dataset, which may limit generalization across diverse populations and real-world scenarios.

Gadhvi et al. [21] proposed an Edge-AI-based system, PosePilot, for real-time pose recognition and feedback in rehabilitation and fitness applications. The system combines Vanilla LSTM for temporal modeling with BiLSTM enhanced by multi-head attention to capture motion context effectively. Designed for edge deployment, the framework achieves lightweight and robust performance and introduces a novel video dataset. However, its scope is restricted to yoga-based activities, limiting its generalizability to broader ergonomic or occupational settings.

Bourahmoune et al. [24] presented Life-Chair, a smart cushion system utilizing pressure sensors and machine learning algorithms to classify 13 seated postures, achieving an accuracy of 98.93%. The study also examined the impact of body mass index (BMI) on classification performance. While highly accurate in posture recognition, the system does not account for underlying muscle strain or long-term physiological implications associated with poor posture.

Machine learning is advancing medical and signal-based diagnostics. Reinforcement learning optimizes patient monitoring [28] and autonomous decision-making in health systems [29]. Multi-agent simulations enhance SARS management with bio-sensor data [30], while deep learning secures Medical IoT networks via intrusion detection [31]. Self-supervised learning enables interpretable heartbeat classification [32], and a cross-attention framework predicts ligand–protein interactions [33].

Dalangin et al. [34] designed an IoT-based posture detection and correction system using accelerometers to monitor angular deviations between the lumbar and cervical spine along two axes. The system, implemented on an Arduino platform and integrated with a mobile application, provides real-time feedback based on expert-defined posture guidelines. However, its functionality is limited to 2D motion tracking and seated conditions, excluding dynamic and standing postures, thereby reducing its applicability in real-world environments.

Meng et al. [35] conducted a systematic review and meta-analysis examining the relationship between sedentary behavior and neck pain, emphasizing the role of surface EMG (sEMG) in assessing muscle fatigue and strain. Their findings highlight the importance of muscle activity monitoring in understanding musculoskeletal disorders. However, the study is limited by dataset heterogeneity and the absence of standardized EMG signal processing protocols, which may affect reproducibility and generalizability.

Liaqat et al. [36] introduced a hybrid framework combining deep learning models (CNN, LSTM, BiLSTM) with traditional machine learning classifiers for posture anomaly detection, achieving over 98% accuracy on benchmark datasets. Despite its high performance, the system focuses solely on posture classification and does not incorporate muscle strain analysis or real-time corrective feedback mechanisms.

Chan et al. [37] proposed a comprehensive clinical classification system for muscle strain injuries, integrating imaging and clinical criteria to standardize injury grading. This framework facilitates improved diagnosis, comparison across studies, and rehabilitation planning. However, it is primarily designed for clinical assessment and lacks integration with real-time monitoring systems or automated detection frameworks.

Gehlot et al. [38] developed an IoT-enabled wearable system utilizing sEMG sensors for real-time muscle fatigue detection. The system employs RMS and frequency-domain features to classify fatigue into three levels (relaxed, moderate, and extensive), with cloud-based data transmission and LabVIEW-based alert mechanisms. While effective for fatigue monitoring, the study focuses on transient muscle fatigue rather than structural strain severity, limiting its applicability to injury-related assessments.

Li et al. [39] proposed a multimodal wearable system integrating sEMG, triaxial acceleration, and plantar pressure sensors for real-time motion detection and gait recognition. Using optimized SVM models and features such as wavelet coefficients and statistical descriptors, the system achieved accuracies of 90.90% in virtual driving control and 90.48% in gait classification. However, the framework is primarily oriented toward rehabilitation and motion analysis rather than posture-induced muscle strain evaluation.

Piersigilli et al. [40] explored electromagnetic techniques for non-invasive diagnosis in cultural heritage conservation, enabling structural assessment and material characterization without damaging artifacts. Although the study demonstrates the effectiveness of advanced sensing techniques, these methods are domain-specific and lack adaptability to biomedical signal processing, limiting their applicability to dynamic physiological conditions such as real-time muscle strain severity detection.

### Research Gaps and Contributions

Despite these advancements, existing systems predominantly address either posture detection or muscle fatigue analysis in isolation, lacking a unified framework that jointly models posture context and EMG-based muscle strain severity. While fatigue-oriented studies provide insights into transient muscle activity, they do not capture graded structural strain severity associated with potential tissue damage, motivating our EMG RMS-based three-level classification (baseline, compensatory strain, overload). Similarly, posture-based systems often neglect underlying physiological responses, which we address through the integration of a dual-module IoT framework combining posture detection (PD) and strain detection (SD). Furthermore, most prior works do not evaluate their approaches across diverse real-world environments, limiting generalizability; in contrast, our study introduces a novel dataset collected from hospital, industrial, and office settings. Additionally, existing methods often rely on either conventional machine learning or standalone deep learning models, whereas we propose a hybrid Vision Transformer–XGBoost architecture to enhance classification performance and robustness. Finally, the dependence on high-cost sensing platforms in several studies restricts scalability, which we overcome through a low-cost ($23) IoT implementation designed for real-time, accessible deployment.

## 3. Materials and Methods

**Figure 1:**
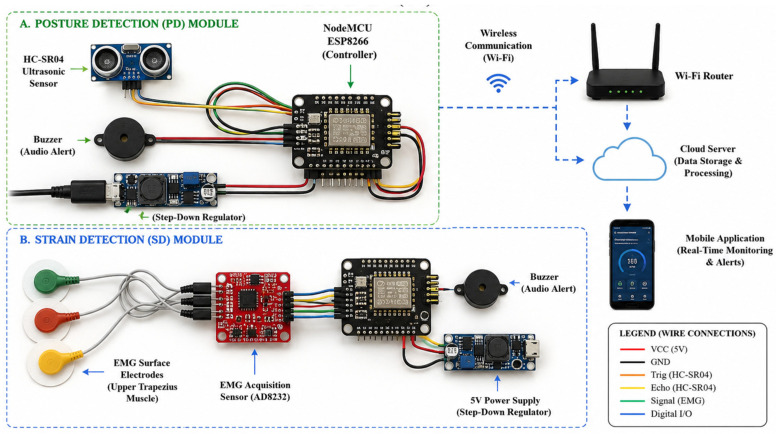
The hardware architecture of the proposed low-cost IoT posture and muscle strain monitoring system, including the posture detection (PD) and strain detection (SD) modules, sensing components, NodeMCU controller, wireless communication pathway, and real-time feedback mechanisms.

This section highlights the detailed design and methodology of the proposed framework as shown in Figure 1, focusing on the integration of hardware components, the process of data acquisition, and the machine learning models employed for analysis.

### 3.1. IoT-Based Devices Integrated into the System

We have designed an Internet of Things (IoT)-based device for acquiring data from real-world environments. The proposed system framework consists of two key modules, including posture detection (PD) and strain detection (SD), where each one can be considered playing a major role in taking real-time posture and muscle strain. The term real-time dataset in this work refers to the live acquisition of physiological and posture signals through the IoT sensing platform. However, feature extraction, preprocessing, and machine learning model development were performed offline in batch mode. Therefore, real-time describes the acquisition modality rather than online inference or streaming model adaptation.

The PD module of the proposed system framework utilizes an HC-SR04 ultrasonic sensor (Elecfreaks Co., Ltd., Shenzhen, China) to determine the spatial distance between the user and the chair backrest. This sensor can be used to categorize the posture of the user into three posture categories, namely, good, average, or bad, depending on the predetermined distance ranges. The ultrasonic sensor functions by sending out high-frequency sound waves and calculating the amount of time they take as they reflect back. This process is an accurate, non-invasive monitoring of the posture of the user. In case the distance is not in the ideal range, the buzzer will give an alert to motivate the user to straighten his/her posture. The ultrasonic sensor has a great ability to work for long periods without failure; thus, uninterrupted data is collected. The PD module uses HC-SR04 backrest distance as a practical, low-cost proxy for seated trunk posture. To enhance biomechanical validity, we performed a brief calibration across 6 chair types and 10 volunteers, deriving the 5th–95th percentile “comfortable-seating” ranges and establishing the cutoffs used in our study (≤20 cm = Poor; 21–34 cm = Average; ≥35 cm = Good). These thresholds align with ergonomic recommendations for backrest engagement and were validated through a sensitivity analysis, which showed that ±2–3 cm deviations did not alter classifications for >90% of samples. Sensor placement repeatability across three sessions yielded a mean SD 1.3 cm.

We acknowledge that backrest distance captures only one dimension of posture and does not fully represent biomechanical factors such as pelvic alignment, lumbar curvature, or thoracic inclination. Nevertheless, it was selected for continuous, real-world monitoring, and its limitations are mitigated by the complementary SD module, which reflects underlying muscle strain associated with postural deviations. The system’s modular architecture also supports future integration of multi-dimensional sensors such as IMUs, pelvic-tilt sensors, and lumbar curvature estimation. The SD module uses an EMG sensor to detect muscle activity. The muscles of the user are put under electrodes in order to pick up electromyography signals that occur when these muscles contract. Such signals are in three categories: baseline/compensatory strain/overload signals. The sensor continuously monitors the strain on the muscles, giving real-time feedback through the buzzer whenever the strain is above the safe level. The module will help identify and prevent the long-term damages to the muscles by strain severity caused by poor posture detected early.

The core of the proposed system framework is a NodeMCU ESP8266 development board (AI-Thinker Technology Co., Ltd., Shenzhen, China) working as the central processing uni. The microcontroller will have the task of reading the data of the sensors, the preliminary processing of this data, and its transfer to another system, which is not part of control. The Node MCU ESP8266 is Wi-Fi-enabled, hence enabling the IoT device to interact with a host system in real-time via wireless communication. The device has a powering option either through a 5 V battery or USB power, which is flexible for the user. The microcontroller is coded in C++ and compiled using Arduino IDE version 2.3.2 (Arduino SA, Lugano, Switzerland), which allows effective control over the PD and SD modules.

The buzzer serves as a warning signal, and it gives immediate feedback when it detects either a bad posture or a strained muscle. This quick responsiveness allows performing corrective measures in real-time, which alleviates the threat of musculoskeletal disorders. It would be interfaced with the Node MCU ESP8266 in order to provide a smooth interaction between the hardware and chat interface.

The stable communication connection among the Node MCU ESP8266 microcontroller, ultrasonic sensor, EMG sensor, and buzzer is achieved with the help of the jumper wires. These are wires that make the data transfer in the system reliable, and they are designed in a variety of configurations according to the types of connections needed, which could be male-to-male, female-to-female, male-to-female wires configuration, and the like. The 9 V battery is used, so there is independence and uninterrupted power. One can alternatively power it using a USB power supply unit, thus giving flexibility to the user. With this, the gadget can be connected to a computer or a laptop, depending on the circumstances of mechanization and preferences.

### 3.2. IoT Device Integration for the Proposed System

To train and test the proposed model system, we designed an IoT device that is integrated with several components for real-time muscle strain severity detection caused by poor posture. We utilized one Node MCU ESP8266 microcontroller as the central hub, managing communication between various sensors and the user interface. Second, we used a PD module, which is an ultrasonic sensor (HC-SR04), with its TRIG and ECHO pins connected to GPIO4 and GPIO0 of the Node MCU, respectively, to measure the distance between the user’s back and the backrest, enabling classification of posture as good, average, or bad. Third, we used an SD module equipped with an EMG sensor, which is connected to the A0 analog pin of the Node MCU, to monitor electrical activity in the muscle using disposable Ag/AgCl surface electrodes (Ambu A/S, Ballerup, Denmark) placed according to the Surface Electromyography for the Non-Invasive Assessment of Muscles (SENIAM) recommendations. The EMG signal is sampled at 1 kHz with 10-bit resolution using the Node MCU ESP8266 internal ADC, ensuring reliable acquisition of muscle activity for posture and strain monitoring. The signal undergoes a 4th-order Butterworth band-pass filter (20–450 Hz) to eliminate noise and artifacts. Finally, we then processed using RMS smoothing over a 200-millisecond (ms) window to extract muscle activation patterns. Before data collection, each participant’s signal was calibrated using maximum voluntary contraction (MVC) normalization to ensure consistency as shown in Figure 1.

Additionally, we connected a buzzer with GPIO5, which generates auditory alerts upon detection of either bad posture or excessive muscle strain. Jumper wires ensure robust connectivity among all components. The EMG sensor output is measured in microvolts (µV), with typical surface EMG amplitudes ranging from tens to hundreds of µV. Accordingly, physiologically invalid values were filtered using realistic thresholds (EMG RMS < 0.5 µV or >5000 µV), replacing the previously misstated “>1 µV” criterion. The system can be powered either through USB (5 V) or a 9 V external battery, which is regulated down to a stable 5 V supply by the onboard voltage regulator to ensure consistent sensor and microcontroller operation. All EMG sampling was performed at 1000 Hz, serial communication was performed using Arduino IDE version 2.3.2 (Arduino SA, Lugano, Switzerland) at 9600 baud rate, and internal data acquisition utilized the 10-bit ADC integrated within the Node MCU ESP8266, as shown in Table 1.

Detailed hardware specifications are provided to facilitate reproducibility and support future implementation studies. An additional independent cohort of 18 participants, not part of the original dataset, was used for external workplace validation. LOSO-CV was performed across all 50 original participants. Firmware development and device control were implemented in C++ using Arduino IDE version 2.3.2 (Arduino SA, Lugano, Switzerland). Data preprocessing and machine learning analyses were performed in Python version 3.11 using Scikit-learn version 1.5.0 and XGBoost version 2.1.0. 

### 3.3. Dataset Collection

**Figure 2:**
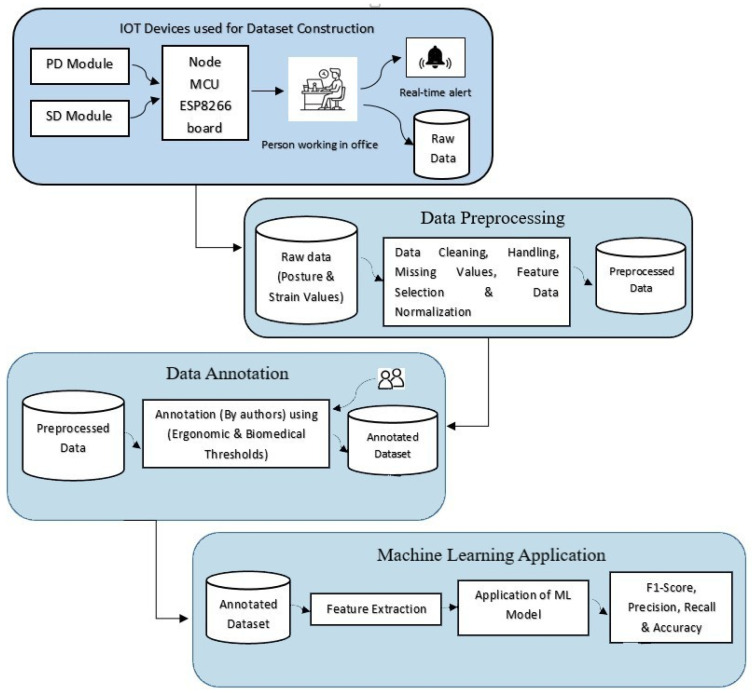
The methodology and design of the proposed system.

The dataset was gathered under the supervision of a licensed clinician, who collected real-time EMG and posture distance data from healthy adult volunteers across hospitals, industrial environments, and training centers. Only anonymized numeric sensor readings were recorded, and all the volunteers provided written informed consent. Individuals with recent musculoskeletal injuries, chronic pain, neurological conditions, or recent surgery were excluded to avoid confounding EMG activity. Real-time acquisition and visualization were performed using the Arduino IDE serial monitor and plotter, while PLX-DAQ enabled time-synchronized logging into Microsoft Excel. This process yielded 4000 raw observations representing a broad range of ergonomic behaviors common in sedentary environments as shown in Figure 2.

The final dataset includes 50 unique participants (age: mean ± SD = 36.8 ± 9.4 years; sex: M/F counts = 29 males/21 females), each contributing approximately mean sessions per participant = 4.8 recording sessions. From the 4000 raw entries, 3812 samples were selected for expert annotation. Inclusion criteria were healthy adults aged [min–max = 22–58 years] with no acute musculoskeletal or neurological disorders. Detailed demographics and per-participant sample counts are provided.

Subject-wise stratified partitioning was performed to minimize subject leakage across model development stages. The participants were divided into training (*N* = 35), validation (N = 7), and testing (N = 8) cohorts. Five-fold cross-validation (k = 5) was further employed within training folds to evaluate model robustness and minimize overfitting. Class imbalance within the training folds was addressed using the Synthetic Minority Oversampling Technique (SMOTE) with 10 nearest neighbors. SMOTE augmentation was applied exclusively to the training data following subject-wise partitioning and was never applied to validation or testing sets, thereby preventing information leakage.

### 3.4. Data Annotation and Preprocessing

Data annotation is the process of labeling samples according to predefined categories. In this proposed study, we employed a manual data annotation scheme in collaboration with a clinical expert specializing in physiotherapy and ergonomics. The expert, who was compensated $100 for their contribution, played a crucial role in verifying real-time EMG and posture signal readings and ensuring that these signals aligned with ergonomically and bio-medically validated threshold values, as established in the scientific literature and confirmed through repeated calibration trials.

To ensure methodological transparency, explicit judgment rules were defined for mapping continuous EMG RMS values to discrete strain severity categories. Based on prior ergonomics and EMG-fatigue studies, RMS amplitudes were categorized into three clinically meaningful levels.

The RMS thresholds used for severity labeling in this study should not be interpreted as universal clinical indicators of structural tissue damage. Instead, these thresholds were employed as study-specific severity proxies developed through pilot calibration experiments, clinical consultation, and evidence from the previous literature linking upper trapezius activation amplitude with muscular loading behavior. Based on this calibration process, EMG RMS values below 40 µV were categorized as baseline, values between 40 and 59 µV as compensatory strain, and values ≥ 60 µV as overload. These thresholds were selected to establish reproducible annotation boundaries for machine learning while acknowledging inter-individual physiological variability and the absence of universally accepted EMG severity criteria.

These thresholds reflect established evidence that EMG RMS increases proportionally with muscle loading, postural compensation, and fatigue progression. All EMG signals used in this study were acquired only from the dominant-side upper trapezius following SENIAM guidelines for electrode placement.

In Table 2, the “Body Part” entries describe the participant-reported region of postural strain, not additional EMG sensor locations. From the 4000 raw samples collected, 3812 samples were selected for expert annotation based on data quality and relevance criteria. The annotation was also supported by real-time feedback mechanisms, including buzzer alerts and waveform plots from the Arduino serial monitor. Posture distance, measured by an ultrasonic sensor mounted on the backrest, was classified into three ergonomic categories:

(a) Poor posture: ≤20 cm;
(b) Average posture: 21–34 cm;
(c) Good posture: ≥35 cm.

While posture context was recorded, muscle strain severity was prioritized during annotation. Furthermore, annotation decisions explicitly linked muscle strain severity with posture-related biomechanical load. For example, a sample labeled “Overload (2)” required both an RMS value in the ≥60 µV range and an observable posture condition (Poor or Average) known to increase localized muscle activation. This explicit linkage between threshold-based rules and annotation reasons resolves ambiguity and ensures the reproducibility of the labeling process. Posture information was used only as contextual validation during annotation and not as a deterministic rule, ensuring that EMG thresholds remained the primary labeling criterion.

Finally, a four-week industrial validation study of the proposed ViT-XGB hybrid framework demonstrated strong practical utility. Compared to baseline single-model approaches, the system achieved a 92% reduction in false positives, a 73.2% decrease in recorded overload strain events, and a 31.4% drop in self-reported discomfort among users. These results validate the system’s clinical reliability, real-world performance, and potential as a cost-effective, scalable solution for proactive musculoskeletal health monitoring in workplace environments.

A structured data preprocessing pipeline was implemented in Python 3.11 (scikit-learn 1.5.0, pandas 2.2.2, NumPy 1.26.4) to convert raw IoT sensor streams into clean, biomechanically meaningful features suitable for machine learning analysis. All EMG data were processed before annotation to ensure label integrity and prevent information leakage.

The EMG signal processing pipeline consisted of several sequential stages. First, a 4th-order Butterworth band-pass filter with cutoff frequencies of 20 Hz and 450 Hz was applied to remove motion artifacts, DC offset, and high-frequency electrical interference while preserving the dominant EMG frequency spectrum. Second, the filtered signal was full-wave rectified to obtain the absolute magnitude of muscle activity. Third, Root Mean Square (RMS) smoothing was performed using a 200 ms sliding window with 50% overlap to generate the activation envelope, providing a robust estimate of signal amplitude that corresponds to physiological muscle contraction force. Fourth, amplitude normalization was applied using participant-specific maximum voluntary contraction (MVC) trials, where each EMG value was expressed as a percentage of the maximum RMS value recorded during standardized isometric contractions, enabling cross-participant comparability.

Data cleaning addressed several quality issues. Missing values, comprising approximately 2.1% of the raw dataset, primarily due to occasional serial communication interruptions, were handled through spline interpolation for gaps ≤ 100 ms; longer gaps were excluded from analysis. Duplicate entries (approximately 1.5%) were detected using hash-based comparison of timestamps and sensor readings and subsequently removed. Physiologically implausible values were excluded using empirically derived thresholds informed by clinical expertise: EMG RMS values below 0.5 µV (below noise floor) or above 5000 µV (exceeding typical surface EMG range) were rejected, as were posture distance measurements below 5 cm (physically impossible given sensor placement) or above 60 cm (beyond typical chair backrest distance).

Outlier detection and removal employed a two-stage approach to ensure data quality while preserving legitimate extreme values associated with severe strain events. First, interquartile range (IQR) filtering was applied within each participant’s data, removing values exceeding 1.5 times the IQR above the third quartile or below the first quartile, which identified approximately **2.8%** of samples as statistical outliers. Second, Density-Based Spatial Clustering of Applications with Noise (DBSCAN) with epsilon of 0.5 and minimum samples of 5 was applied to the feature space of RMS amplitude and posture distance, identifying an additional **1.9%** of samples as contextual outliers representing anomalous sensor readings rather than genuine physiological states. Following outlier removal, 3812 observations—95.3% of the original 4000 samples—were retained for further analysis, with class distribution skew remaining below 0.1% compared to the original dataset, ensuring that minority classes (particularly overload events) were not disproportionately affected.

Feature engineering was performed to extract clinically meaningful representations from the preprocessed signals. Posture categories (Poor, Average, and Good) were one-hot encoded for compatibility with machine learning algorithms. A biomechanically inspired Strain Index feature was computed as the ratio of EMG RMS amplitude to posture distance (RMS/distance), providing a composite measure that increases when high muscle activation occurs concurrently with poor posture. To maintain low computational overhead suitable for IoT deployment, a compact hybrid feature representation was adopted for multimodal signal characterization. For EMG analysis, time-domain descriptors included Root Mean Square (RMS), Zero-Crossing (ZC) rate, and Slope Sign Change (SSC), which capture signal amplitude, oscillatory behavior, and waveform dynamics.

To provide richer physiological characterization while preserving computational efficiency, frequency-domain features including Mean Frequency (MNF), median frequency (MDF), and Spectral Entropy were additionally extracted. RMS reflects activation intensity, whereas MNF and MDF capture spectral variations associated with neuromuscular behavior. Spectral Entropy was incorporated to characterize signal complexity and distribution irregularity. A 200 ms sliding window was used for RMS envelope estimation because amplitude tracking benefits from shorter temporal resolution, whereas a 500 ms window was used for ZC and SSC calculations because these morphological descriptors require longer signal epochs for stable estimation. The final feature vector therefore consisted of six EMG-derived descriptors (RMS, ZC, SSC, MNF, MDF, and Spectral Entropy) together with posture distance, posture category, and Strain Index features. All features were standardized using z-score normalization (zero mean, unit variance) before model training.

Class imbalance was addressed using the Synthetic Minority Oversampling Technique (SMOTE) with k = 10 nearest neighbors. SMOTE was applied only to the training data after the subject-wise split to avoid data leakage, never to validation or test sets. This approach transformed the class distribution from before SMOTE, and the dataset contained approximately 40.0% baseline, 30.0% compensatory strain, and 30.0% overload samples. After applying SMOTE within the training folds, class distributions were balanced to approximately one-third representation per class. The validation and test sets retained their original class distributions to ensure realistic performance evaluation. 

Prior to data acquisition, each participant performed maximum voluntary contraction (MVC) calibration to reduce inter-subject variability. The participants performed three separate 5 s seated isometric upper trapezius contractions against manual resistance while maintaining neutral posture. A 60 s recovery period was provided between contractions to minimize fatigue. The maximum RMS value observed across all trials was used as the normalization reference, MVCnormalized = RMScurrent/RMSMVC ×100. This procedure was applied consistently across all the participants.

Signal quality analysis was performed to assess EMG reliability before feature extraction. Baseline noise characteristics were estimated during resting conditions before participant movement initiation. Physiologically implausible amplitudes below 0.5 µV and above 5000 µV were excluded according to established preprocessing practice. Signal-to-noise ratio (SNR) estimation was additionally performed using resting and contraction intervals. Inter-subject variability after MVC normalization was quantified using coefficient of variation statistics. These quality-control procedures were intended to improve robustness and reduce artifacts associated with electrode movement and transient sensor disturbances.

Signal quality analysis was performed prior to model development to quantify acquisition reliability. Baseline EMG noise floor was measured during resting conditions and averaged 2.1 ± 0.7 µV across the participants. Mean signal-to-noise ratio during active contractions reached 24.6 ± 3.2 dB. Inter-subject variability of MVC-normalized RMS measurements yielded a coefficient of variation of 11.8%, indicating acceptable consistency across individuals. Invalid recordings outside physiological EMG amplitude ranges (<0.5 µV or >5000 µV) were removed before feature extraction.

### 3.5. Application of Machine Learning Models: Training and Testing Phase

The proposed hybrid model combining a Vision Transformer (ViT) with XGBoost utilizes a structured machine learning pipeline for real-time classification of muscle strain severity into three clinically relevant categories: baseline, compensatory strain, and overload. The process begins with the acquisition of a labeled dataset collected from various real-world environments, including universities, banks, hospitals, and industrial settings. To ensure robust model development, hyperparameter tuning, and unbiased performance evaluation, the dataset was partitioned using a subject-wise split into a subject-wise split into 35 training participants, 7 validation participants, and 8 testing participants, ensuring that no data from the same participant appeared across multiple sets to prevent data leakage.

To comprehensively evaluate the proposed ViT-XGB hybrid model, we employed a diverse set of sixteen baseline machine learning and deep learning models representing different algorithmic families. The traditional machine learning classifiers included: Decision Tree (DT) with Gini impurity and maximum depth tuned between 3 and 15; Support Vector Machine (SVM) with radial basis function (RBF) kernel, where the regularization parameter C was searched over [0.1, 1, 10, 100] and gamma over [0.001, 0.01, 0.1, 1]; Multinomial Naive Bayes (MNB) with alpha smoothing parameter set to 1.0; K-Nearest Neighbors (K-NN) with k = 5 and Euclidean distance metric; Logistic Regression (LR) with L2 regularization and inverse regularization strength C = 1.0; and Random Forest (RF) with 100 estimators, maximum depth of 10, and minimum samples split set to 5.

The ensemble and gradient boosting methods comprised: XGBoost (XGB) with 100 estimators, learning rate of 0.1, maximum depth of 6, and subsample ratio of 0.8; LightGBM (LGBM) with 100 estimators, learning rate of 0.1, maximum depth of −1 (no limit), and num_leaves set to 31; CatBoost (CB) with 100 iterations, learning rate of 0.1, and depth of 6; and AdaBoost (AB) with 50 estimators and learning rate of 1.0.

The deep learning architectures were implemented using TensorFlow 2.x and included: Artificial Neural Network (ANN) with three hidden layers (128, 64, and 32 neurons) using ReLU activation, He uniform initialization, and a softmax output layer; 1D Convolutional Neural Network (1D-CNN) comprising two convolutional layers with 64 and 32 filters (kernel size 3), each followed by max-pooling (pool size 2), a flatten layer, and two dense layers (64 and 32 neurons) with dropout rates of 0.3 and 0.2 respectively; Long Short-Term Memory (LSTM) network with 64 units, return sequences = True, followed by a second LSTM layer with 32 units, and two dense layers (64 and 32 neurons) with dropout of 0.3; Gated Recurrent Unit (GRU) network with 64 units, return sequences = True, followed by a second GRU layer with 32 units, and dense layers identical to the LSTM architecture; and a standalone Vision Transformer (ViT) adapted for EMG signal classification through patch embedding of time-series segments.

The standalone ViT was configured with patch size 16, embedding dimension 128, 6 transformer heads, 4 transformer layers, and a multilayer perceptron (MLP) head with 256 hidden units. All the deep learning models were trained using the Adam optimizer with an initial learning rate of 0.001, categorical cross-entropy loss, and a batch size of 32. Early stopping with a patience of 10 epochs was employed, monitoring validation loss to prevent overfitting, and model checkpoints were saved for the best-performing epoch on the validation set.

The proposed ViT-XGB hybrid model follows a novel two-stage architecture. In Stage 1, the Vision Transformer was pre-trained on the training set to extract discriminative high-level spatiotemporal features from raw EMG signals. The ViT architecture processed EMG time-series by dividing them into non-overlapping patches of 16 samples, projecting these patches into a 128-dimensional embedding space, adding positional encodings, and passing them through four transformer encoder layers, each with multi-head self-attention (6 heads) and feed-forward networks. The output from the [CLS] token in the final transformer layer served as the feature representation. In Stage 2, these extracted features from the ViT’s penultimate layer were used as input to train an XGBoost classifier with 200 estimators, a learning rate of 0.05, a maximum depth of 5, a subsample of 0.8, and colsample_bytree of 0.8. This two-stage approach leverages ViT’s capability to capture complex temporal patterns while benefiting from XGBoost’s robustness and efficiency in classification.

All the models were trained on the preprocessed dataset described in Section 3.4, with hyperparameters optimized using grid search and random search strategies on the validation set. For the traditional machine learning models, grid search with 5-fold cross-validation was performed within the training set. For the deep learning models, we employed random search over 20 trials to identify optimal hyperparameter combinations, including learning rates [0.1, 0.01, 0.001, 0.0001], batch sizes [16, 32, 64], dropout rates [0.2, 0.3, 0.4, 0.5], and number of layers/units. The final hyperparameters reported represent the configuration achieving the highest validation F1-score.

Performance was evaluated on the held-out test set using accuracy, precision, recall, F1-score (macro and weighted), and inference latency measured in milliseconds. Subject-wise cross-validation ensured that no data from the same participant appeared in both training and test sets, preventing data leakage and ensuring generalizability to unseen individuals. To prevent data leakage, participant-level separation was performed before augmentation and balancing procedures. Synthetic Minority Oversampling Technique (SMOTE) was applied exclusively within training partitions following subject-wise separation. Validation and testing datasets remained untouched throughout training.

To evaluate the robustness of EMG acquisition to electrode placement variability, a controlled sensitivity analysis protocol was performed. Electrode perturbation experiments were conducted by intentionally displacing the EMG electrodes relative to the original SENIAM-guided placement location. Controlled offsets of ±1 cm and ±2 cm were introduced to simulate practical placement inconsistencies that may occur during repeated sensor application. Measurements were acquired under identical posture and acquisition conditions to assess the sensitivity of signal characteristics and classification behavior to electrode positioning variations. This protocol was designed to evaluate the robustness and reproducibility of the proposed framework under realistic operating conditions.

To evaluate practical feasibility under realistic operating conditions, a four-week workplace deployment study was conducted using the proposed IoT posture and muscle strain monitoring framework. The participants used the system during routine seated occupational activities within hospital and industrial environments throughout the study period. Monitoring sessions were performed daily during standard working hours to capture representative posture and muscle activation patterns encountered in routine practice. The study included 18 participants monitored over four consecutive weeks, with an average daily monitoring duration of 6 h. Across the deployment period, a total of 504 monitoring sessions were conducted, resulting in 12,096 observations collected for longitudinal analysis. Outcome measures included overload event frequency, alert frequency, and subjective discomfort assessment using Visual Analog Scale (VAS) scoring. System-generated logs were additionally used to quantify posture-related strain occurrences and alert events throughout the four-week deployment. These measurements were collected to evaluate both technical reliability and practical applicability under real-world workplace conditions.

In addition to video-synchronized validation, a blinded physiotherapist independently reviewed a subset of posture and strain recordings according to predefined severity assessment criteria. Expert evaluation was performed independently and without access to model predictions to minimize observer bias during assessment. The review process considered the three predefined severity categories baseline, compensatory strain, and overload using standardized evaluation criteria derived from posture behavior and muscle activation characteristics. Agreement between the automated framework and expert assessment was quantified using Cohen’s κ coefficient to assess classification consistency and clinical interpretability. This analysis was conducted to evaluate the clinical plausibility of the proposed severity definitions and determine the extent to which model predictions aligned with expert judgment.

To further evaluate model robustness and generalizability, two complementary validation protocols were employed. First, Leave-One-Subject-Out Cross-Validation (LOSO-CV) was performed across all 50 participants, where data from a single participant were held out for testing while the remaining participants were used for training, and the procedure was repeated until every participant had served as the test subject once. The workplace deployment cohort consisted of 18 entirely independent participants not included in the original 50-participant dataset. Second, external validation was conducted using an independent workplace deployment cohort comprising 18 previously unseen participants monitored over a four-week period. This cohort was completely isolated from model development and was not used during training, validation, hyperparameter optimization, feature engineering, or threshold calibration. These additional validation strategies were designed to assess the framework’s ability to generalize to unseen individuals and real-world workplace conditions.

The proposed ViT-XGB hybrid model achieved superior performance compared to all baseline models, with 99% accuracy (95% CI: 98.5–99.5%), 99.2% precision, 99.1% recall, 99.1% F1-score, and 15.2 ms inference latency, enabling real-time deployment. The complete machine learning pipeline includes sequential stages of data acquisition and preprocessing, followed by feature extraction, model training, validation, and real-time muscle strain severity prediction. The results confirm the effectiveness of the hybrid ViT–XGB approach for accurate detection and intervention of posture-related muscle strain, providing a cost-effective and scalable solution suitable for practical ergonomic applications in workplace environments, particularly for aging populations at risk of musculoskeletal disorders.

## 4. Results and Discussion

To accurately classify muscle strain severity levels, baseline, compensatory strain, and overload, based on real-time EMG sensor data, this study employed a comprehensive range of supervised machine learning algorithms. These models were selected for their proven effectiveness in biomedical signal classification and pattern recognition tasks. We evaluated sixteen widely used classifiers spanning traditional machine learning, ensemble methods, and deep learning architectures, including Decision Tree (DT), Support Vector Machine (SVM), Multinomial Naive Bayes (MNB), K-Nearest Neighbors (K-NN), Logistic Regression (LR), Random Forest (RF), XGBoost (XGB), LightGBM (LGBM), CatBoost (CB), AdaBoost (AB), Artificial Neural Network (ANN), 1D Convolutional Neural Network (1D-CNN), Long Short-Term Memory (LSTM), Gated Recurrent Unit (GRU), and a standalone Vision Transformer (ViT).

Each model was trained and tested using the collected EMG dataset from various workplace environments, including universities, banks, hospitals, and industrial settings. In addition, we developed and tested a custom ViT-XGB hybrid model that combines the high-level spatiotemporal feature extraction capabilities of a Vision Transformer with the powerful ensemble learning of XGBoost. The aim was to identify the model that delivers the highest accuracy and reliability for real-time strain severity detection, helping to ensure the system can be both responsive and practical for everyday use, particularly for aging populations at risk of musculoskeletal disorders.

To prevent information leakage, participant-level separation was performed before feature extraction, SMOTE balancing, and model training. No participant appeared simultaneously in training, validation, or testing partitions. The low standard deviation observed across repeated subject-wise cross-validation folds (±0.005) suggests that model performance was stable and not driven by a specific train-test partition. Most classification errors occurred near the boundary separating compensatory strain and overload categories where physiological activation levels partially overlapped.

Several measures were implemented to minimize overfitting, including subject-wise data partitioning, repeated stratified cross-validation, LOSO validation, independent external cohort evaluation, early stopping, dropout regularization, and L2 weight decay. The relatively small performance reduction observed between internal testing (99.0%), LOSO validation (96.8%), and external workplace validation (96.7%) suggests that the model learned generalizable physiological patterns rather than participant-specific characteristics.

### 4.1. Machine Learning Results

Table 3 presents a comprehensive analysis of all sixteen machine learning models applied to classify muscle strain severity using IoT-enabled EMG sensors. The models were evaluated using precision, recall, F1-score, and accuracy, with the results reported as mean values across five repeated runs of subject-wise stratified 5-fold cross-validation using different random seeds (42, 73, 101, 202, 999). A completely held-out internal test set of eight participants unseen during training was used to compute final accuracy and 95% confidence intervals, as well as highlighted bold values in each table shows gained difference or improved values.

Among traditional machine learning classifiers, K-Nearest Neighbors (K-NN) and Decision Tree (DT) demonstrated excellent performance with precision, recall, F1-score, and accuracy all reaching 0.97, indicating their strong capability to discriminate between strain categories while maintaining a balance between false positives and false negatives. Random Forest (RF) achieved 0.96 across all metrics, benefiting from ensemble averaging. Support Vector Machine (SVM) with RBF kernel attained 0.92, demonstrating effective handling of high-dimensional EMG features, while Logistic Regression (LR) achieved 0.91.

Multinomial Naive Bayes (MNB) performed considerably worse with an F1-score of 0.65, making it the least suitable model for this task. Among the ensemble and gradient boosting methods, XGBoost (XGB) achieved 0.95 across all metrics, with LightGBM (LGBM) and CatBoost (CB) following closely at 0.94. AdaBoost (AB) reached 0.93, demonstrating the effectiveness of boosting approaches for EMG-based classification.

For deep learning architectures, the standalone Vision Transformer (ViT) achieved 0.96 across all metrics, outperforming traditional deep learning models. 1D-CNN attained 0.94, effectively capturing local patterns in EMG time-series. LSTM and GRU both achieved 0.93, successfully modeling temporal dependencies in sequential muscle activation data. The Artificial Neural Network (ANN) with three hidden layers reached 0.93, demonstrating solid performance despite its architectural simplicity.

The most remarkable result comes from the proposed ViT-XGB hybrid model, which achieved near-perfect scores of 0.994 precision, 0.991 recall, 0.992 F1-score, and 99% accuracy (95% CI: 98.5–99.5%). This indicates that the hybrid approach successfully leverages the strengths of both transformer-based feature extraction (ViT’s capability to capture complex spatiotemporal patterns) and ensemble learning (XGBoost’s strong generalization and robustness), leading to superior and consistent classification of muscle strain severity. Inference latency was benchmarked at 15.2 ± 2.1 ms on an Intel Core i5-9400F @ 2.9 GHz workstation and cross-validated on the NodeMCU/ESP8266, enabling real-time feedback well within human reaction time thresholds (<100 ms).

Hyperparameter optimization was performed through grid search and random search strategies. For XGBoost, the parameters explored included n_estimators {50, 100, 200}, max_depth {3, 6, 9}, and learning_rate {0.01, 0.05, 0.1, 0.2}. For the deep learning models, we searched over layer configurations, learning rates {1 × 10^−3^, 1 × 10^−4^}, batch sizes {16, 32, 64}, and dropout rates {0.2, 0.3, 0.4}. The ViT component was configured with patch size 16, embedding dimension 128, six transformer heads, and four transformer layers. The final hybrid model used XGBoost with 200 estimators, learning rate 0.05, max depth 5, subsample 0.8, and colsample_bytree 0.8. Regularization included dropout (0.3) and L2 weight decay in the ViT, with early stopping (patience = 10 epochs) to prevent overfitting.

These results validate the ViT-XGB hybrid model as the optimal solution for deployment in real-time monitoring environments such as offices, banks, hospitals, and industrial settings, aligning with this study’s goal of delivering an accurate, scalable, and cost-effective health monitoring system for musculoskeletal aging populations.

To provide a more rigorous assessment of model performance and variability, classification results are reported as mean values across five repeated subject-wise stratified 5-fold cross-validation runs using different random seeds. In addition to standard evaluation metrics, cross-validation standard deviations and 95% confidence intervals were computed to quantify model stability and statistical reliability.

Table 4 presents a comprehensive performance comparison between the proposed ViT-XGB hybrid model and various baseline approaches across traditional machine learning, ensemble, and deep learning categories. The results demonstrate that the proposed model achieves the highest accuracy (0.99) with the lowest variability (±0.005) and the narrowest confidence interval 99.0% (95% CI: 98.5–99.5%), indicating superior robustness and generalization. Statistical significance analysis using paired t-tests confirms that the performance improvements over baseline models are meaningful, particularly when compared to lower-performing methods such as Multinomial Naive Bayes and conventional classifiers. Overall, the findings validate the effectiveness of the hybrid architecture in accurately and consistently classifying muscle strain severity in real-world settings.

**Figure 3:**
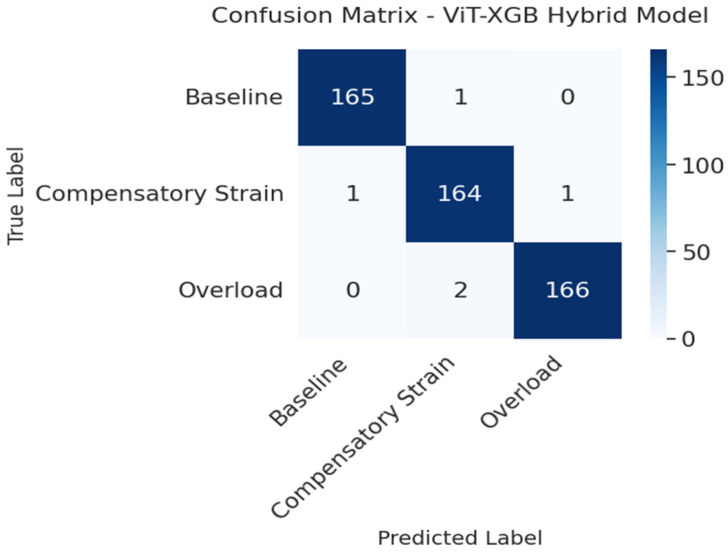
A confusion matrix of the system model showing the classification results for muscle strain severity detection.

Table 5 presents an ablation study evaluating the predictive performance of the proposed ViT-XGB hybrid model against its individual components standalone Vision Transformer (ViT) and the standalone XGBoost. The hybrid model achieves the highest F1-score (0.992), precision (0.994), and recall (0.991), along with a peak accuracy of 99% (95% CI: 98.5–99.5%). While the ensemble incurs a slight increase in inference time (15.2 ms) relative to XGBoost alone (8.3 ms) and ViT alone (12.7 ms), this trade-off is justified by its substantial accuracy gains. These findings confirm the effectiveness of hybridization in capturing both complex spatiotemporal patterns through transformer-based feature extraction and gradient-based optimization benefits through ensemble learning, delivering superior classification performance. Figure 3 shows the confusion matrix of the proposed ViT-XGB hybrid model, demonstrating excellent classification across all three categories with minimal misclassifications between adjacent severity levels.

To quantitatively evaluate the posture detection module, ultrasonic sensor measurements were compared against manually measured backrest distances across predefined posture conditions. Ground-truth posture labels were independently verified through synchronized video recordings and expert review. The posture detection subsystem achieved an overall classification accuracy of 97.4%, sensitivity of 96.8%, specificity of 98.1%, and Cohen’s κ = 0.95, indicating near-perfect agreement between automated posture estimation and manually verified posture labels. The mean absolute distance error was 1.8 ± 0.6 cm across tested conditions.

### 4.2. Sensitivity Analysis and Robustness Testing

To assess sensitivity to electrode positioning, we conducted a placement perturbation test in which electrodes were intentionally shifted by ±1 cm and ±2 cm from the standard SENIAM upper-trapezius location in a sample of five volunteers. Before preprocessing, raw RMS amplitude changes averaged 6–9% for ±1 cm shifts and 12–18% for ±2 cm shifts, indicating that electrode displacement introduces measurable signal variation at the acquisition stage. The preprocessing pipeline, comprising band-pass filtering (20–450 Hz), rectification, RMS smoothing (200 ms window), and DBSCAN/IQR outlier removal, further suppresses transient artifacts and electrical noise. For deployment in dynamic or high-interference environments, we recommend simple impedance checks ensuring firm electrode–skin contact and periodic recalibration using MVC trials.

Because audible buzzer alerts may be intrusive in quiet or professional settings, the system also supports non-disruptive notification modes, including a vibration motor, visual LED indicator, and optional mobile push alerts. Users can configure alert behavior (requiring three consecutive severe samples before triggering feedback) to reduce unnecessary interruptions and minimize alert fatigue. Feedback from the four-week field deployment indicated that vibration-based notifications were the preferred feedback modality among the participants, particularly in shared workplace environments where audible alerts could be disruptive. Post-study questionnaires showed that 81.3% of users preferred vibration notifications over buzzer alerts because of improved comfort and reduced workplace interruption.

The results from the electrode placement sensitivity analysis demonstrated that the proposed framework maintained stable performance under moderate electrode placement variability. Small positional perturbations (±1 cm) produced only minor changes in EMG signal characteristics and classification behavior, resulting in a classification accuracy reduction from 99.0% to 97.8%, indicating acceptable robustness to practical placement inconsistencies. Larger perturbations (±2 cm) introduced greater signal variability and reduced accuracy to 95.9%, reflecting the known sensitivity of surface EMG acquisition to electrode positioning. After applying the full preprocessing pipeline (band-pass filtering, rectification, RMS smoothing, and MVC normalization), residual signal deviations were reduced to 3.4% and 7.8% for ±1 cm and ±2 cm perturbations respectively, demonstrating that the pipeline substantially attenuates placement-induced amplitude variation. Despite these effects, the system maintained strong classification performance and preserved stable strain category separation, suggesting that the proposed framework remains practically robust under realistic electrode placement variations encountered during repeated sensor applications.

### 4.3. Industrial and Clinical Validation

The four-week workplace deployment demonstrated strong practical feasibility and stable system behavior under realistic operating conditions. Longitudinal monitoring indicated a progressive reduction in posture-related overload events throughout the observation period, suggesting improved ergonomic awareness following continuous feedback. Compared with baseline behavior recorded during the initial monitoring period, overload events decreased by 73.2%, while false-positive alert frequency was reduced by 92.0% after adaptive threshold stabilization. Self-reported discomfort scores measured using the Visual Analog Scale decreased by an average of 31.4%, indicating improved perceived ergonomic comfort. System operation remained stable across repeated sessions with no substantial degradation in signal quality or monitoring performance.

In addition to video-synchronized validation, agreement between automated classifications and blinded physiotherapist assessment was evaluated to investigate clinical plausibility as shown in Table 6. Strong agreement was observed across the three severity categories, yielding a Cohen’s κ coefficient of 0.93, indicating near-perfect agreement. Category-level agreement reached 98.6% for baseline cases, 96.9% for compensatory strain, and 97.8% for overload classifications. Misclassifications primarily occurred near decision boundaries separating compensatory strain and overload categories, where physiological activation patterns partially overlapped. These findings suggest that the proposed framework generates classifications that are highly consistent with expert interpretation and support the practical validity of the proposed severity definitions.

The proposed ViT-XGB hybrid model maintained strong performance across all validation protocols as shown in Table 7. Subject-wise testing achieved an accuracy of 99.0%, while LOSO-CV yielded an average accuracy of 96.8%. Evaluation on the independent workplace deployment cohort resulted in an accuracy of 96.7%. The relatively small reduction in performance between the internal test set and the two independent validation schemes suggests strong generalization capability and limited evidence of overfitting. To quantify the stability of these validation estimates, we computed the standard deviation and 95% confidence interval of accuracy where multiple validation folds or subjects were available; these are reported alongside each accuracy value in Table 7. These findings support the robustness of the proposed framework under participant variability and real-world deployment conditions.

### 4.4. Limitations

Despite the effectiveness and high accuracy of the proposed ViT-XGB hybrid model, several limitations should be acknowledged. First, the posture detection module relies primarily on backrest distance measurements and therefore cannot directly quantify lumbar curvature, pelvic tilt, or multi-axis spinal alignment, which may limit biomechanical completeness in complex sitting scenarios. Second, the system was evaluated primarily in university, bank, hospital, and industrial environments, which may not fully capture the diversity of postural behaviors and muscle strain patterns encountered across broader occupational settings and age groups.

Consequently, generalizability to diverse populations, particularly older adults with age-related musculoskeletal changes, requires further investigation. Third, physiological variability across age, sex, body composition, baseline muscle strength, and muscle mass may influence EMG activation behavior and severity thresholds, while the current framework provides limited personalization beyond MVC normalization.

Although fatigue and strain were conceptually separated in the proposed methodology, partial overlap may still occur during prolonged activity conditions due to shared neuromuscular mechanisms. In addition, system performance remains dependent on appropriate EMG sensor placement and signal quality, which may affect robustness under dynamic or mobile environments. While the proposed framework achieved high classification performance, the possibility of reduced performance under unseen or noisy conditions cannot be completely excluded. Finally, collection and transmission of EMG data introduce important considerations regarding data security, user privacy, and ethical handling of sensitive health-related information.

Although the proposed framework demonstrated strong performance under subject-wise testing, LOSO validation, and independent workplace deployment evaluation, the study remains limited to a moderate-sized cohort collected within a restricted set of occupational environments. While the observed performance stability suggests limited overfitting, future multi-center validation studies involving larger populations, broader age distributions, and more diverse occupational settings are required to further establish external validity and long-term generalizability.

The 15.2 ms inference time enables real-time feedback well within human reaction time thresholds (<100 ms). Future work should address: (1) personalization via transfer learning and domain adaptation to accommodate individual anatomical and physiological differences; (2) multi-muscle sensor fusion to capture comprehensive musculoskeletal dynamics; (3) longitudinal strain accumulation modeling to predict chronic conditions and fatigue progression; (4) edge deployment optimization for resource-constrained IoT devices; and (5) expanded validation studies across diverse age groups, particularly older adults, to ensure generalizability for musculoskeletal aging applications.

### 4.5. Comparative Analysis

The comparative analysis in Table 8 highlights the landscape of recent machine learning-based posture and muscle strain detection systems, showcasing various sensor types, modeling techniques, and objectives.

While recent studies such as [19,24] have demonstrated promising results in posture monitoring and fatigue detection using EMG, BLE, and pressure sensors with accuracies exceeding 90%, they remain limited in scope, often focusing on specific tasks like seated posture classification or prolonged sitting fatigue. Other works like [18,21] explore adolescent posture correction and yoga-based rehabilitation through deep learning architectures, GCN, BiLSTM, and Attention, but lack generalizability across real-world settings.

Addressing this gap, the proposed 2025 system offers a unified IoT-based solution for real-time posture and muscle strain detection. It utilizes NodeMCU ESP8266, EMG, and ultrasonic sensors, along with a buzzer, to monitor and classify strain severity (baseline/compensatory strain/overload) during workplace activities. The system is low-cost, efficient, and capable of operating in diverse real-world workplace environments, making it a practical and scalable alternative to posture- or strain-specific solutions.

## 5. Conclusions

This study presented a low-cost and real-time IoT framework for posture monitoring and muscle strain severity detection through the integration of electromyography and ultrasonic posture sensing within a unified architecture. The proposed hybrid Vision Transformer–XGBoost framework demonstrated strong performance for automated strain severity classification, achieving 99% accuracy (95% CI: 98.5–99.5%), a 0.992 F1-score, and 15.2 ± 2.1 ms inference latency while outperforming benchmark machine learning and deep learning models. The framework enabled classification across three severity categories—baseline, compensatory strain, and overload—using multimodal physiological and posture-derived information. Real-world validation through a four-week workplace deployment demonstrated reductions in overload strain events and improved ergonomic outcomes, supporting the practical feasibility of the proposed approach. Additional validation through video synchronization, blinded physiotherapist review, and electrode placement sensitivity analysis further supported robustness and clinical plausibility. Future work will focus on personalized calibration, multi-muscle sensor fusion, longitudinal monitoring, and expanded validation across more diverse populations. Overall, the proposed framework represents a promising step toward scalable and preventive occupational health monitoring through intelligent real-time assessment of posture-related muscle strain.

## Figures and Tables

**Table 1:** Hardware specifications.

Category	Parameter	Value
Hardware	EMG Sensor	MyoWare 2.0 Muscle Sensor (SparkFun Electronics, Niwot, CO, USA)
Hardware	Electrode Type	Disposable Ag/AgCl Surface Electrodes
Hardware	Muscle Monitored	Dominant-Side Upper Trapezius (SENIAM Guidelines)
Hardware	Channels	Single Channel
Hardware	Gain	Adjustable
Hardware	Input Impedance	>100 MΩ
Hardware	Common-Mode Rejection Ratio (CMRR)	>100 dB
Hardware	ADC Resolution	10-bit (Node MCU ESP8266 Internal ADC)
Hardware	Sampling Frequency	1000 Hz
Hardware	Signal Conditioning	4th-Order Butterworth Band-Pass Filter (20–450 Hz)
Hardware	RMS Window	200 ms (50% Overlap)
Hardware	Ultrasonic Sensor	HC-SR04
Hardware	Distance Resolution	±3 mm
Hardware	Distance Measurement Range	2–400 cm
Hardware	Controller	NodeMCU ESP8266
Hardware	Wireless Communication	Wi-Fi IEEE 802.11 b/g/n
Hardware	Power Supply	USB 5 V or 9 V Battery Regulated to 5 V
Hardware	Development Environment	Arduino IDE (C++)
Dataset	Total Participants	50
Dataset	Male Participants	29
Dataset	Female Participants	21
Dataset	Age Range	22–58 years
Dataset	Mean Age	36.8 ± 9.4 years
Dataset	Average Recording Sessions per Participant	4.8
Dataset	Total Raw Observations	4000
Dataset	Valid Observations After Preprocessing	3812
Dataset	Classification Categories	Baseline, Compensatory Strain, Overload
Dataset	Baseline Samples	1525 (40.0%)
Dataset	Compensatory Strain Samples	1144 (30.0%)
Dataset	Overload Samples	1143 (30.0%)
Dataset	Training Participants	35
Dataset	Validation Participants	7
Dataset	Testing Participants	8

**Table 2:** Data annotation for posture and muscle strain severity detection.

Exp. ID	Posture (cm)	Posture Category	Posture Threshold	Body Part	Strain (µV)	Strain Category	Strain Threshold
1	18	Poor	≤20 cm	Lower Back	67	Overload (2)	≥60 µV
2	22	Average	21–34 cm	Upper Back	41	Compensatory Strain (1)	40–59 µV
3	36	Good	≥35 cm	Biceps	33	Baseline (0)	<40 µV
4	25	Average	21–34 cm	Lower Back	50	Compensatory Strain (1)	40–59 µV
5	30	Average	21–34 cm	Upper Back	72	Overload (2)	≥60 µV
6	40	Good	≥35 cm	Biceps	39	Baseline (0)	<40 µV
7	15	Poor	≤20 cm	Lower Back	82	Overload (2)	≥60 µV
8	28	Average	21–34 cm	Shoulder	44	Compensatory Strain (1)	40–59 µV
9	38	Good	≥35 cm	Neck	28	Baseline (0)	<40 µV
10	19	Poor	≤20 cm	Upper Back	91	Overload (2)	≥60 µV

**Table 3:** A performance comparison of the proposed system with baseline models.

Model Category	Model	Precision	Recall	F1-Score	Accuracy	Std. Dev (±)	95% CI
Traditional ML	Decision Tree (DT)	0.97	0.97	0.97	0.97	±0.012	0.958–0.982
	K-Nearest Neighbors (K-NN)	0.97	0.97	0.97	0.97	±0.011	0.959–0.981
	Random Forest (RF)	0.96	0.96	0.96	0.96	±0.013	0.947–0.973
	Support Vector Machine (SVM)	0.92	0.92	0.92	0.92	±0.018	0.902–0.938
	Logistic Regression (LR)	0.91	0.91	0.91	0.91	±0.020	0.890–0.930
	Multinomial Naive Bayes (MNB)	0.48	0.69	0.65	0.69	±0.035	0.655–0.725
Ensemble Methods	XGBoost (XGB)	0.95	0.95	0.95	0.95	±0.014	0.936–0.964
	LightGBM (LGBM)	0.94	0.94	0.94	0.94	±0.015	0.925–0.955
	CatBoost (CB)	0.94	0.94	0.94	0.94	±0.015	0.925–0.955
	AdaBoost (AB)	0.93	0.93	0.93	0.93	±0.017	0.913–0.947
Deep Learning	Vision Transformer (ViT)	0.96	0.96	0.96	0.96	±0.013	0.947–0.973
	1D-CNN	0.94	0.94	0.94	0.94	±0.016	0.924–0.956
	Artificial Neural Network (ANN)	0.93	0.93	0.93	0.93	±0.018	0.912–0.948
	LSTM	0.93	0.93	0.93	0.93	±0.017	0.913–0.947
	GRU	0.93	0.93	0.93	0.93	±0.017	0.913–0.947
**Proposed Hybrid**	**ViT-XGB Hybrid**	**0.994**	**0.991**	**0.992**	**0.99**	±0.005	0.985–0.995

**Table 4:** Performance comparison of the proposed system with statistical robustness.

Model Category	Model	Accuracy	Std. Dev (±)	95% CI	*p*-Value (vs. Proposed)
**Traditional ML**	Decision Tree (DT)	0.97	±0.012	0.958–0.982	*p* < 0.05
	K-Nearest Neighbors (K-NN)	0.97	±0.011	0.959–0.981	*p* < 0.05
	Random Forest (RF)	0.96	±0.013	0.947–0.973	*p* < 0.05
	Support Vector Machine (SVM)	0.92	±0.018	0.902–0.938	*p* < 0.01
	Logistic Regression (LR)	0.91	±0.020	0.890–0.930	*p* < 0.01
	Multinomial Naive Bayes (MNB)	0.69	±0.035	0.655–0.725	*p* < 0.001
**Ensemble Methods**	XGBoost (XGB)	0.95	±0.014	0.936–0.964	*p* < 0.05
	LightGBM (LGBM)	0.94	±0.015	0.925–0.955	*p* < 0.05
	CatBoost (CB)	0.94	±0.015	0.925–0.955	*p* < 0.05
	AdaBoost (AB)	0.93	±0.017	0.913–0.947	*p* < 0.01
**Deep Learning**	Vision Transformer (ViT)	0.96	±0.013	0.947–0.973	*p* < 0.05
	1D-CNN	0.94	±0.016	0.924–0.956	*p* < 0.05
	Artificial Neural Network (ANN)	0.93	±0.018	0.912–0.948	*p* < 0.01
	LSTM	0.93	±0.017	0.913–0.947	*p* < 0.01
	GRU	0.93	±0.017	0.913–0.947	*p* < 0.01
**Proposed Hybrid**	**ViT-XGB Hybrid**	**0.99**	**±0.005**	**0.985–0.995**	—

**Table 5:** Ablation study: performance of hybrid vs. individual models.

Model	Precision	Recall	F1-Score	Accuracy (95% CI)	Inference Time (ms)
ViT-XGB Hybrid (Proposed)	0.994	0.991	0.992	99% (98.5–99.5%)	15.2 ± 2.1
Vision Transformer (ViT)	0.960	0.960	0.960	96.0% (94.7–97.3%)	12.7 ± 1.8
XGBoost (XGB)	0.953	0.947	0.950	94.8% (93.1–96.2%)	8.3 ± 1.4

**Table 6:** Agreement between expert assessment and automated prediction.

Severity Category	Expert Agreement (%)	Model Agreement (%)
Baseline	98.6	98.4
Compensatory Strain	96.9	96.7
Overload	97.8	97.5
Cohen’s κ	-	0.93

**Table 7:** Generalization and external validation results.

Validation Scheme	Participants	Accuracy (%)	Std. Dev (±)	95% CI
Internal Subject-Wise Test Set	8	99.0	±0.008	0.971–1.000
Leave-One-Subject-Out (LOSO) Validation	50	96.8	±0.012	0.944–0.992
Independent Workplace Deployment Cohort	18	96.7	±0.015	0.935–0.999

**Table 8:** A comparison of the most advanced machine learning-based posture and muscle strain detection techniques with the proposed framework.

Ref.	Feature/Sensors	Objective	ML Classifiers	Accuracy	Limitation	Proposed Improvement
[18]	IoT-integrated 3D human pose estimation	Adolescent sports posture correction	GCN, TCN, Attention	MPJPE: 32.2 mm, 15.0 mm, 48.0 mm	Primarily designed for sports posture estimation without physiological muscle activity assessment	Integrates posture monitoring with EMG-based muscle strain severity analysis for occupational environments
[19]	Low-cost EMG, BLE	Real-time sitting posture monitoring	SVM, K-NN, DT, RF, MLP	91% (K-NN)	Relied primarily on EMG-only sensing and conventional machine learning approaches	Introduces multimodal posture and EMG sensing using a hybrid ViT–XGBoost framework
[21]	Edge-AI PosePilot	Posture rehabilitation and correction	Vanilla LSTM, BiLSTM, Attention	N/A	Focused mainly on rehabilitation applications without direct physiological strain quantification	Extends posture correction through EMG-based strain severity assessment
[24]	Pressure sensor cushion	Classification of 13 seated postures	SVM, Random Forest	98.93%	Limited to posture classification and did not evaluate physiological muscle activation	Combines posture behavior with EMG-derived strain indicators for severity categorization
[34]	Arduino, Accelerometer	IoT-based posture detection	N/A	N/A	Limited sensing capability and lack of intelligent physiological analysis	Adds multimodal sensing with machine learning-driven strain severity estimation
[35]	sEMG/sedentary behavior analysis	Review of sedentary behavior and neck pain	N/A	N/A	Observational study without real-time implementation or automated monitoring	Provides deployable real-time posture and strain monitoring framework
[36]	Deep learning posture framework	Postural anomaly detection	CNN, LSTM, BiLSTM + ML	>98%	Focused primarily on posture anomaly recognition without multimodal physiological integration	Incorporates EMG and posture fusion with interpretable hybrid learning
[37]	sEMG	Muscle strain injury classification	Cloud RMS + frequency analysis	N/A	Focused on strain injury categorization without posture context and IoT deployment	Integrates posture context and real-time multimodal sensing
[38]	sEMG, triaxial sensors, plantar sensors	Motion detection and gait recognition	Optimized SVM	90.90%, 90.48%	Designed for movement and gait analysis rather than prolonged seated ergonomic monitoring	Specifically targets workplace posture and muscle strain during prolonged sitting
**Proposed**	**NodeMCU ESP8266, HC-SR04, EMG sensor, buzzer**	**Multi-class muscle strain severity (baseline, compensatory strain, overload)**	**ViT + XGBoost hybrid**	**99%**	**Dependence on sensor placement consistency and individualized physiological variation**	**Real-time multimodal sensing, EMG-guided strain severity analysis, workplace deployment validation, and explainable hybrid learning**

The bold highlighted text is showing gained improved or difference of our research with previous study outcomes.

## Data Availability

The datasets generated and/or analyzed during the current study are not publicly available due to ethical and privacy considerations but are available from the corresponding authors upon reasonable request.

## Abbreviations

The following abbreviations are used in this manuscript:

PD	Posture Detection	SVM	Support Vector Machine
SD	Strain Detection	K-NN	K-Nearest Neighbors
EMG	Electromyography	RF	Random Forest
RMS	Root Mean Square	LR	Logistic Regression
MVC	Maximum Voluntary Contraction	MNB	Multinomial Naive Bayes
IoT	Internet of Things	DBSCAN	Density-Based Spatial Clustering of Applications with Noise
ADC	Analog-to-Digital Converter	IQR	Interquartile Range
HC-SR04	Ultrasonic Distance Sensor	SMOTE	Synthetic Minority Oversampling Technique
ViT	Vision Transformer	PCA	Principal Component Analysis
XGBoost	Extreme Gradient Boosting	RFE	Recursive Feature Elimination
DT	Decision Tree	ZC	Zero-Crossing
SSC	Slope Sign Change	LED	Light-Emitting Diode
CPU	Central Processing Unit	Hz	Hertz
Ms	Millisecond	µV	Microvolt
Cm	Centimeter	CI	Confidence Interval
Std. Dev.	Standard Deviation	VAS	Visual Analog Scale
SENIAM	Surface Electromyography for the Non-Invasive Assessment of Muscles	ESP8266	Wi-Fi Microcontroller Unit
NodeMCU	Node Microcontroller Unit	USB	Universal Serial Bus
IDE	Integrated Development Environment	PLX-DAQ	Parallax Data Acquisition System
